# Press tack needle stimulation for blunt chest trauma: a randomized double-blind control trial

**DOI:** 10.1093/icvts/ivac158

**Published:** 2022-06-07

**Authors:** Pei-Yu Kao, Bernice Lottering, Ting-Yu Lu, Wen-Chao Ho, Yu-Sen Lin, Tzu-Min Huang, Chien-Kuang Chen, Jian-Xun Chen, Yu-Chen Lee, Fang-Pey Chen, Eyal Ben-Arie

**Affiliations:** Division of Thoracic Surgery, Department of Surgery, China Medical University Hospital, Taichung, Taiwan; Institute of Traditional Medicine, School of Medicine, National Yang-Ming University, Taipei, Taiwan; Graduate Institute of Acupuncture Science, China Medical University, Taichung, Taiwan; Division of Thoracic Surgery, Department of Surgery, China Medical University Hospital, Taichung, Taiwan; Department of Public Health, China Medical University, Taichung, Taiwan; Division of Thoracic Surgery, Department of Surgery, China Medical University Hospital, Taichung, Taiwan; Division of Thoracic Surgery, Department of Surgery, China Medical University Hospital, Taichung, Taiwan; Division of Thoracic Surgery, Department of Surgery, China Medical University Hospital, Taichung, Taiwan; Division of Thoracic Surgery, Department of Surgery, China Medical University Hospital, Taichung, Taiwan; Graduate Institute of Acupuncture Science, China Medical University, Taichung, Taiwan; Department of Acupuncture, China Medical University Hospital, Taichung, Taiwan; Institute of Traditional Medicine, School of Medicine, National Yang-Ming University, Taipei, Taiwan; Center for Traditional Medicine, Taipei Veterans General Hospital, Taipei, Taiwan; Graduate Institute of Acupuncture Science, China Medical University, Taichung, Taiwan

**Keywords:** Blunt chest trauma, Acupuncture, Rib fracture, Press tack needles, Analgesia

## Abstract

**OBJECTIVES:**

Blunt chest trauma is often associated with severe pain, reduced lung function and decreased sleep quality. This study aims to investigate the immediate and long-term effect of acupuncture on these factors using a randomized control double-blind design.

**METHODS:**

A total of 72 patients were randomized into 2 groups: treatment group (press tack acupuncture) and control group (press tack placebo). The face rating scale, numerical rating scale (NRS), portable incentive spirometer and Verran Snyder-Halpern sleep scale were measured at baseline, immediately after the intervention, and at the 4th day, with 2-weeks and 3-months follow-ups.

**RESULTS:**

There were no significant changes between the groups at the baseline measurements, with the exception of hypertension comorbidity. Immediately after the intervention and on the 4th day follow-up, the patients in the treatment group showed a significantly lower face rating scale when compared to the control (*P* < 0.05). There were no significant changes in any of the other measurements between the groups (*P* > 0.05). Subgroup analysis revealed that the NRS for turn over on the 4th day was reduced significantly in the treatment group of patients without lung contusion (*P* < 0.05). For patients without pleural drainage, cough NRS in the treatment group was significantly reduced in the 2-week follow-up (*P* < 0.05).

**CONCLUSIONS:**

This study showed that press tack acupuncture effects on pain reduction were inconclusive. However, future studies on the effect of acupuncture on blunt chest trauma patients are needed.

**Clinical trial registration:**

clinicaltirl.gov: NCT04318496.

**Figure:**
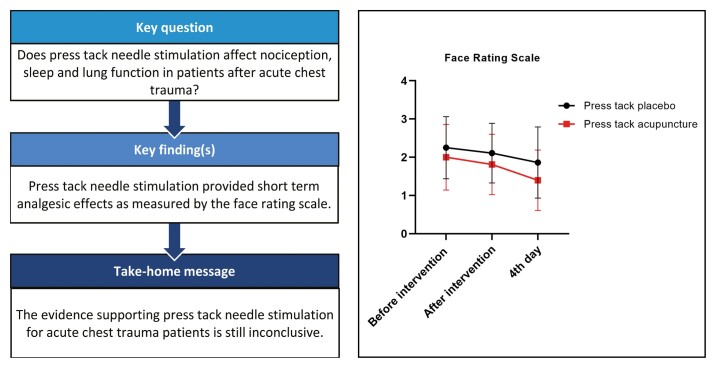

## INTRODUCTION

Blunt chest trauma (BCT) is defined by a blunt injury to the rib cage, whereby the injury can vary in its severity from a local tissue injury to lethal injuries [1]. The literature showed that patients with >2 rib fractures will experience worse outcomes and may require more hospitalization, intensive care unit admissions and more mechanical ventilation [2]. As a result of BCT episode, patients will experience severe pain that persists for a long duration after the acute injury [3, 4]. This discomfort affects the patient’s ability to perform common daily life activities and disturbs the patient’s sleep [5, 6]. Since it is reported that many regeneration and healing processes occur during sleep, sleep disturbance can lead to delayed recovery and longer periods of disability and pain [7]. As a result of the injury to the local tissue, the pulmonary tissue or the pain sensation, normal breathing abilities might be impaired [8].

Apart from life-saving interventions and rib fracture fixation, the treatment options offered to patients suffering from BCT consist of analgesics and nerve block medication [9]. Nerve blocks are usually only applied to severe level 3 patients [2]. Analgesics are commonly offered to patients from all 3 levels along with rest [9]. However, analgesics are often carrying a number of side effects, can possibly lead to addiction and do not assist in the regeneration processes [10]. Older patients and patients with renal insufficiency are at higher risk to suffer from the detrimental side effects associated with the long-term use of analgesics [11]. A possible alternative for the use of analgesics is acupuncture. Acupuncture analgesia has been clearly demonstrated in several animal studies and clinical trials [12, 13]. In theory, acupuncture can also increase blood flow to the injured tissue and assist the natural regeneration processes [14, 15]. The effect of acupuncture on BCT was previously investigated in 1 clinical trial that found evidence of an analgesic effect that lasted 6 hours [16]. Further credible reports on the function of acupuncture in the treatment of BCT patients are warranted. This study hypothesizes that significant differences will be found between the verum and placebo acupuncture groups in terms of analgesic effect, sleep quality and breathing abilities.

## METHODS

### Ethics statement

The study followed the ethical requirements of the institutional review board of China Medical University Hospital (CMUH109-REC1-002). The study protocol was also registered on clinicaltirl.gov (NCT04318496). All patients included in the study signed an informed consent form.

### Design and setting

This was a double-blind randomized control trial involving 2 equally allocated groups from a single medical centre in Taiwan. The study took place in the China Medical University Hospital and its out-patients clinics. The study duration was from March 2020 until June 2021. A detailed study protocol was published on: https://journals.lww.com/md-journal/Fulltext/2021/05070/Acupuncture_for_blunt_chest_trauma__A_protocol_for.33.aspx.

### Participants

The study included 72 patients that suffered from acute BCT. The patients visited the hospital emergency department, followed by hospitalization or outpatient clinic for follow-up. Patients were enrolled after visiting the above-mentioned locations. The patient recruitment was granted upon filling out an informed consent form, of which the details are provided in the study protocol.

### Inclusion criteria

- Age 20–80 years old.
- Patients who have chest trauma as described by themselves or based on medical chart records within 1 week of the injury.
- Injury severity score (ISS) is <18 points.
- Body mass index <30.

### Exclusion criteria

- Sternal fracture.
- ISS is ≥18 points.
- History of intercostal nerve injury.
- History of cardiovascular disease.
- History of chronic lung disease.
- Significant lung mass or chest deformity noted in the chest plain film.

### Randomization allocation and blinding

Patients were randomized using a computer-based simple randomization (SPSS software, ver. 22) into one of the groups with 1:1 ratio. A random number (1–72) was written down in sequentially numbered, opaque, sealed envelopes filled with a set of press tack needles (for the treatment group) or press tack placebos without a needle (for the control group) that are identical in their outer presentation. All patients, doctors, acupuncturists and outcome assessors were blind to the patient group allocation throughout the study. The acupuncturist was asked not to closely examine the needles when applying them on the patient. In addition, the press tack placebo contains a small metal object covered with clear plastic that is placed in the location where the needle is placed. This gives the illusion that the press tack placebo contains a needle as well.

### Intervention

After patient allocation, both groups received the allocated intervention (press tack needles or placebos) (PYONEXΦ0.20 mm × 0.6 mm made by Seirin Corporation) on the following acupoints: GB 36 (WaiQiu), GB 34 (YangLingQuan), ST 36 (ZuSanLi), LI 4 (HeGu), LU 7 (LieQue) and TH 5 (WaiGuan) bilaterally. Detailed descriptions of the intervention are available in the published study protocol. The intervention in both groups was administered on the day of enrolment. The press tacks were kept for 4 days and were withdrawn in the follow-up session in the hospital/clinic by a qualified medical professional for blinding purposes. The retention time interval was suggested as the press tack needle’s manufacturer. The patients were prescribed analgesic and/or muscle relaxant medications as needed.

### Outcome measurements

The main outcome measurements include the levels of pain measured on a numerical rating scale (NRS), on the following activities: rest, deep breath, cough and turn-over movements. In the NRS, the patients report their level of pain in a scale between 0 and 10 points: 0 points indicate no pain and 10 points indicate a very severe pain. In addition, pain and the overall feelings of the patient were measured by the face rating scale (FRS) 0–5, filled in by the patient. In the FRS, the patients chose one of 5 faces, a score of 0 indicate no pain and a good feeling (smiley face) and a score of 5 indicate severe pain and a bad feeling (unhappy and crying face). Secondary measurements include the portable incentive spirometer (Tri-flow). The Tri-flow test the exhalation ability of the patient, the Tri-flow score is ranging between 0 and 1200 cc per/sec. The Verran Snyder-Halpern sleep scale, and the total dose of intravenous analgesic medication used (in total number of patients require intravenous analgesics use in each group). The Verran Snyder-Halpern sleep scale measure the patient sleep ability in 3 domains: sleep disturbance, sleep effectiveness and sleep supplementation, A higher score in sleep effectiveness and a decrease in sleep disturbance indicate desirable sleep goals. An increase in sleep supplementation may indicate a need for sleep during the day.

The measurements were obtained physically on the first session and at the 4th day follow-up (primary). At the time point of 2 weeks and 3 months after enrolment (secondary), telephone calls measured the NRS in rest, deep breath, cough, turn over movements and side effects.

### Sample size calculation

The study sample size was based on Ho *et al.* [16] study that previously investigated the use of acupuncture on acute chest trauma. The study findings were used to calculate this current trial sample size as generated by G∗power version 3.1.9.2 software. After considering 95% power, an effect size of 1.124365 in independent *t*-test was determined. The calculation revealed that the study required at least 36 patients, 18 in each group. However, when considering the fact that Ho *et al.* study included 29 patients in each group, and adding a predicted 20% drop out rate, the current study required 36 patients in each group for a total of 72 patients.

### Statistical analysis

The SPSS software ver. 22.0 (SPSS Inc, Chicago, IL, USA) was used to implement the statistical analysis for this study. To determine the normal distribution, the Shapiro–Wilk test was used. When the data distribution was normal, the independent *t* test was used to compare between the 2 groups. For non-normal distribution, the Mann–Whitney *U* test was used to compare between the 2 groups. For categorical data, the Chi-squared test was used. The study analysis followed an intent to treat concept. The study missing data ranges between 0 and 30.6% in some outcomes (all 3 months NRS—30.6%, all 2 weeks NRS—26.4%, 4th day FRS—9.7%, immediate after FRS—8.3%, cough 4th day NRS—6.9%, cough baseline NRS—5.6%, baseline FRS—4.2%, body turn 4th day NRS, deep breath baseline NRS, deep breath 4th day NRS—2.8%, rest 4th day NRS, deep breath immediate after NRS, body turn baseline NRS and body turn immediate after NRS—1.4%); the missing data pattern fits the missing completely at random pattern (Little's MCAR test: Chi-square = 835.191, DF = 833, sig. = 0.472). Missing data were imputed by series mean when the data had normal distribution. In the cases of non-normal distribution, the missing data were imputed by series median. The original data without missing data imputation is available in Supplementary Material, Table S1.

## RESULTS

**Figure 1:**
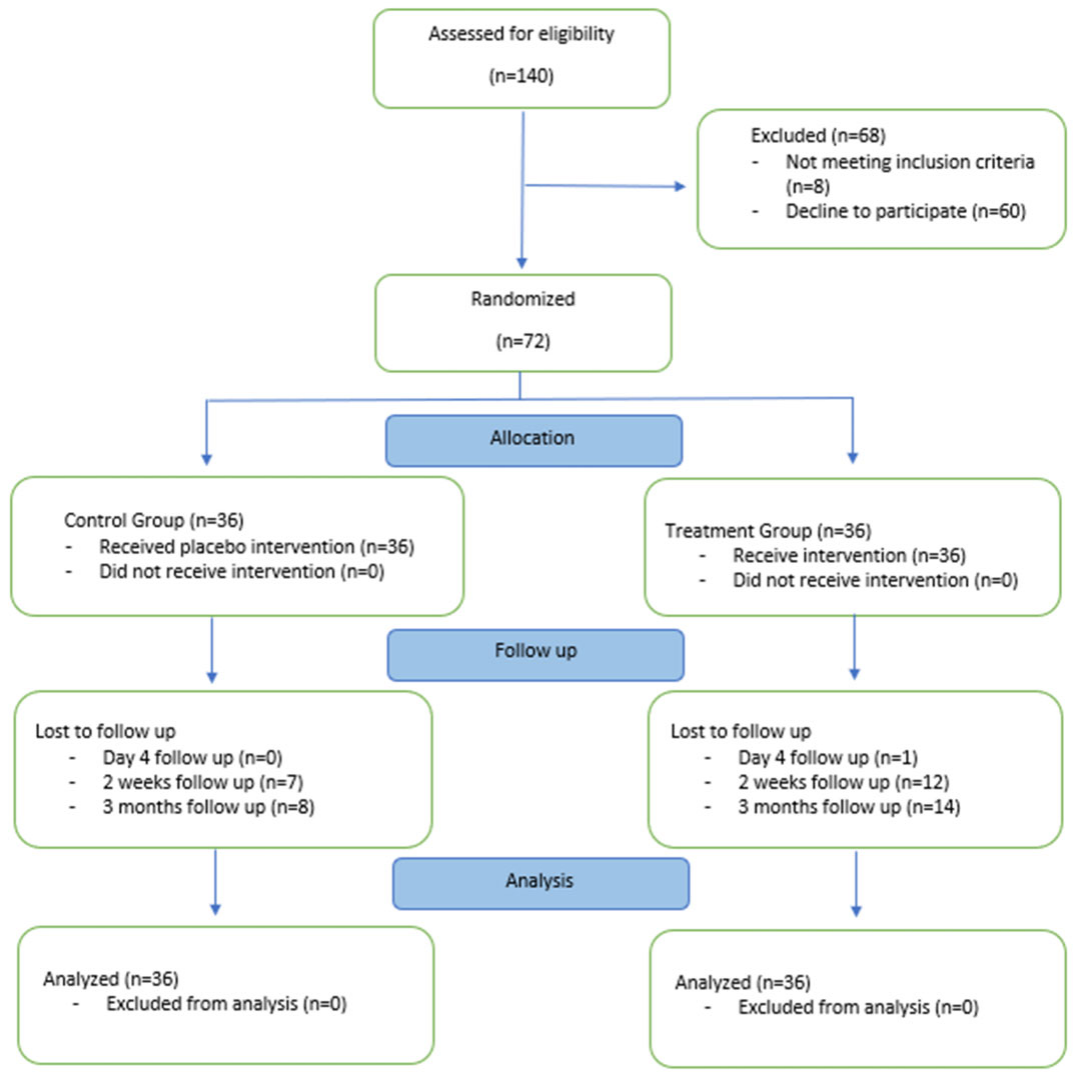
The figure describes the study processes. As for the intent-to-treat analysis, all the included patients were analysed. Missing values were imputed.

The study enrolled 72 patients, 36 in each group. In the treatment group, 1 patient did not come back for the 4th day follow-up, 19 patients missed the 2-weeks telephone follow-up and 22 patients missed the 3-months telephone follow-up. For the primary outcomes, missing values were imputed. There are no significant differences in gender, age, ISS score, number of rib fractures, haemothorax and pneumothorax. However, there was a significant difference observed between the groups in the number of hypertension patients, with 3 patients in the treatment group versus 12 patients in the control group (*P* = 0.009) (Table 1 and Fig. 1). Furthermore, there were no significant differences between the groups in NRS scale for rest, deep breath, cough and body turn over movements, as well as the Tri-flow, face rating, overall sleep disturbance, effectiveness and supplementation in VHS scale (*P* > 0.05 for all measurements) on the baseline measurements.

**Table 1:** Baseline characteristics

	Treatment group (*n* = 36)	Control group (*n* = 36)	*P*-Value
Age, mean ± SD	49.50 ± 16.10	52.03 ± 14.70	0.489
Gender (F/M)	15/21	12/24	0.465
ISS, mean ± SD	7.50 ± 4.49	8.58 ± 4.74	0.323
Rib fractures no., mean ± SD	2.89 ± 2.29	3.08 ± 2.12	0.709
Days from injury, mean ± SD	2.94 ± 3.00	2.22 ± 1.48	0.201
Haemothorax, *n* (%)	8 (22.2)	7 (19.4)	0.772
Pneumothorax, *n* (%)	7 (19.4)	9 (25.0)	0.571
Lung contusion, *n* (%)	3 (8.3)	2 (5.6)	1.000
Hospitalization, *n* (%)	25 (69.4)	29 (80.6)	0.276
Needed chest drainage tube, *n* (%)	3 (8.3)	7 (19.4)	0.173
Comorbidities, *n* (%)			
Stroke	3 (8.3)	0 (0.0)	0.239
Cancer	0 (0.0)	3 (8.3)	0.239
Hypertension	3 (8.3)	12 (33.3)	0.009^*^
Diabetes	6 (16.7)	6 (16.7)	1.000
Others	7 (19.4)	10 (27.8)	0.405

The table describes the baseline characteristics of the patients at the time of patient enrolment.

* Significant differences between the groups *P* < 0.05.

F: female; ISS: injury severity score; M: male; SD: standard deviation.

After treatment, there was no significant difference observed between the groups throughout the follow-up periods as determined by the NRS score of rest and mobile status immediately after the intervention, or on the 4th day, 2-weeks and 3-months follow-ups (*P* > 0.05, respectively). The FRS (measuring general pain) was significantly decreased immediately in the treatment group 1.81 ± 0.79 vs 2.11 ± 0.78 in the control group (*P* = 0.044) and also significantly lower on the follow-up session on the 4th day in the treatment group 1.40 ± 0.79 vs 1.86 ± 0.93 in the control (*P* = 0.038) (Table 2). A number of patients refused to measure the pain deep breath, pain cough and pain turning over due to ‘too much pain’ (Supplementary Material, Table S2); those missing values were also imputed. Subgroup analysis was performed in the following groups: patients without lung contusion and patients without pleural drainage. The NRS score on body turning and FRS score on the 4th day were significantly lower in the treatment group compared to the control group in the patients without lung contusion subgroup (*P* = 0.049, 0.037 respectively). The NRS score on cough on the 2-weeks follow-up and FRS on the 4th day follow-up were significantly lower in the treatment group compared to the control group in the patients without pleural drainage subgroup (*P* = 0.039 and 0.042, respectively, Table 3).

**Table 2:** Main outcomes

	Treatment group (*n* = 36)	Control group (*n* = 36)	*P*-Value
Face rating scale, mean ± SD			
Baseline	2.00 ± 0.86	2.25 ± 0.81	0.273
Immediately after	1.81 ± 0.79	2.11 ± 0.78	0.044^*^
Day 4	1.40 ± 0.79	1.86 ± 0.93	0.038^*^
NRS score, mean ± SD			
Pain rest			
Baseline	3.13 ± 2.10	3.47 ± 2.63	0.759
Immediately after	2.78 ± 2.25	3.18 ± 2.46	0.511
Day 4	1.94 ± 1.71	2.42 ± 1.98	0.320
2 weeks	1.97 ± 1.80	2.36 ± 2.00	0.400
3 months	0.32 ± 1.05	0.40 ± 0.94	0.190
Pain deep breath			
Baseline^a^	4.37 ± 2.23	4.57 ± 2.27	0.715^**^
Immediately after^a^	3.88 ± 2.06	4.17 ± 2.58	0.600^**^
Day 4	2.88 ± 1.75	3.38 ± 2.29	0.464
2 weeks	1.82 ± 1.86	2.39 ± 2.44	0.580
3 months	0.38 ± 1.03	0.29 ± 1.03	0.686
Pain cough			
Baseline	6.35 ± 2.44	6.71 ± 2.69	0.500
Immediately after^a^	5.94 ± 2.33	6.30 ± 2.71	0.550^**^
Day 4^a^	4.62 ± 2.19	5.25 ± 2.87	0.298^**^
2 weeks	2.96 ± 1.84	3.94 ± 2.51	0.059
3 months	0.51 ± 1.22	0.49 ± 1.47	0.745
Pain body turn			
Baseline	6.82 ± 2.48	7.28 ± 2.27	0.392
Immediately after	6.56 ± 2.68	6.13 ± 2.93	0.583
Day 4	4.63 ± 2.41	5.57 ± 2.32	0.067
2 weeks	3.21 ± 2.27	4.07 ± 2.59	0.165
3 months	0.50 ± 1.16	0.46 ± 0.93	0.705

The table describes the main outcomes for this study at the different follow-ups. Missing data were imputed by series median and analysed by the Mann–Whitney *U* test.

NRS: numeric rating scale; SD: standard deviation.

a Missing data were imputed by series mean.

* Significant differences between the groups *P* < 0.05.

** *P*-value was analysed by the independent *t* test.

**Table 3:** Subgroup analysis

	Treatment	*n*	Control	*n*	*P*-Value
Pain body turn (NRS), mean ± SD					
Patients without lung contusion					
Baseline	6.84 ± 2.53	32	7.27 ± 2.26	34	0.446
4th day	4.44 ± 2.33	32	5.54 ± 2.33	34	0.049^*^
2 weeks	2.98 ± 2.06	32	4.04 ± 2.66	34	0.131
Patients without pleural drainage					
Baseline	6.74 ± 2.50	33	7.10 ± 2.20	30	0.577
4th day	4.44 ± 2.27	*33*	5.52 ± 2.29	30	0.060
2 weeks	3.27 ± 2.34	*33*	4.20 ± 2.74	30	0.201
Pain cough (NRS), mean ± SD					
Patients without lung contusion					
Baseline	6.34 ± 2.57	32	6.81 ± 2.55	34	0.481
4th day^a^	4.57 ± 2.29	32	5.32 ± 2.84	34	0.243^**^
2 weeks	2.97 ± 1.95	*32*	3.91 ± 2.58	34	0.123
Patients without pleural drainage					
Baseline	6.33 ± 2.47	33	6.62 ± 2.67	30	0.638
4th day^a^	4.60 ± 2.22	33	5.45 ± 2.78	30	0.183^**^
2 weeks	2.94 ± 1.89	*33*	4.13 ± 2.60	30	0.039^*^
Face rating scale, mean ± SD					
Patients without lung contusion					
Baseline	2.00 ± 0.92	32	2.26 ± 0.83	34	0.286
4th day	1.39 ± 0.82	*32*	1.88 ± 0.95	34	0.037^*^
Patients without pleural drainage					
Baseline	1.97 ± 0.88	33	2.17 ± 0.70	30	0.358
4th day	1.35 ± 0.81	*33*	1.83 ± 0.91	30	0.042^*^

The table describes the subgroup analysis of predetermined groups. Missing data were imputed by series median and analysed by the Mann–Whitney *U* test.

NRS: numeric rating scale; SD: standard deviation.

a Missing data were imputed by series mean.

* Significant differences between the groups *P* < 0.05.

** *P*-Value was analysed by the independent *t* test.

There was no significant difference between the groups in Tri-flow measurements (*P* > 0.05 respectively) nor in the overall sleep disturbance, effectiveness and supplementation in VHS scale (*P* > 0.05 respectively, Table 4 and Supplementary Material, Table S3).

**Table 4:** Secondary measurements

	Treatment group	*n*	Control group	*n*	*P*-Value
Tri-flow, mean ± SD					
Baseline	653.78 ± 356.33	36	700.72 ± 309.15	36	0.552
Immediately after	775.39 ± 342.95	36	818.75 ± 298.81	36	0.569
Day 4	920.63 ± 221.60	35	913.11 ± 215.84	36	0.807
Hospital stay (in days), mean ± SD	5.64 ± 2.23	25	5.76 ± 2.53	29	0.944
Outpatient department		11		7	
Patients need IV analgesics, *n* (%)	9 (25.0)	36	12 (33.3)	36	0.437

The table describes the secondary measurements. Missing data were not imputed (original data were used).

IV: intravenous; SD: standard deviation; Tri-flow: portable incentive spirometer.

In regard to blinding, the acupuncturist guessed the type of needles wrongly 41.4% of the treatments. The patients guessed 56.1% of the needle type wrongly (Supplementary Material, Table S4).

No adverse events were reported in this study.

## DISCUSSION

This is the first double-blind randomized control trial that investigated the effect of press tack needle stimulation on BCT patients. The study found a significant general pain reduction according to FRS immediately after the intervention and on the 4th day follow-up period in the treatment group when compared to the control group, but no significant reduction in NRS measurements. In a subgroup analysis, the NRS score on body turning and FRS on day 4 was significantly reduced in the treatment group when compared to the control group in patients without lung contusion. And there was also a significant reduction in the pain measured by the NRS cough score on the 2 weeks, and on the 4th day FSR follow-up in patients without pleural drainage.

Pain scales are commonly used in medical practice. The most commonly used pain scales are NRS, visual analogue scale (VAS), verbal rating scale and face pain scale. To choose which one to use depends on the compliance of scale in combination with the characteristics of the pathology. VAS was reported to display more difficulties in practice and is suggested to be used in combination with other pain scales [17]. In a systematic review, the NRS displayed superior application and was easier to use when compared to VAS and verbal rating scale [18]. The FRS can describe, in addition to the overall pain sensation, pain interference and pain unpleasantness [19]. In our study, we found a significant reduction in the FRS both immediately after the interventions and on the 4th day follow-up measurements in the treatment group when compared to the control group. This indicates that the overall feeling and ability to tolerate pain were improved after receiving the verum press tack acupuncture.

With regard to the pain control policy in BCT patients, multimodal methods are regularly suggested [9]. However, aggressive pain management approaches, including epidural analgesia, and intrapleural and intercostal nerve blocks are mostly applied to high severity or multiple rib fracture patients. Treatment options for lower ISS score patients are limited to oral analgesic and self-rehabilitation. Regardless of the patient’s severity, the level of pain will be high in all kinds of BCT patients. Fabricant *et al.* [20] found that after an average of 2 months from the injury, 66% of rib fracture patients still use opioids and 31% of the patients still presented with isolated rib fractures. Of the isolated rib fracture patients, 64% suffered long-term pain and 66% suffered continued disability (2 months or longer) [20]. Marasco *et al.* found a long-term reduction in quality of life in multiple rib fracture patients, lasting up to 2 years, accompanied by a poor return to work rates [21]. In the present study, we included different variety of patients in age, gender and severity. In subgroup analyses, the press tack needle reduced pain significantly in the 4th day measurements with regard to turning over pain in the group without pulmonary contusion, and in the 2 weeks measurements for coughing pain in the group without pleural drainage. This might provide another treatment option in the multimodal pain control policy for patients with less severe BCT.

Age is an important element in BCT mortality and morbidity [22]. The incidence of pneumonia and mortality in elderly patients with rib fractures is double, even if presented as minor trauma at the time of admission. Our study did not find a correlation between age and pain relief by acupuncture.

Acupuncture use on BCT was not sufficiently investigated until now. In a clinical trial conducted by Ho *et al.*, acupuncture displayed a rapid pain reduction in deep breath, cough and turning over movements that last 6 h after acupuncture with 2.5− cm− long and 30− gauge filiform needles in the treatment of rib fracture patients [16]. A small cohort study also showed acupuncture-related pain reduction after 6 months, although these changes might be attributed to the length of time from injury [23]. A case report also showed a good analgesic effect for chest trauma following auricular acupuncture [24]. Compared to previous studies, our trial provided a double-blind design that was yet to be implemented in this field. Our trial was designed based on the convenience for both patients and medical practitioners. In traditional acupuncture, daily or multiple sessions of acupuncture are often required. The analgesic effects experienced are often reduced after the removal of the filiform needles [16]. In the present study, retention of press tack needles for 4 days was proven to be safe with no reported complications or infection, and single intervention was needed. Press tack needle application is indicated by shallow penetration with finer needle insertion in anatomy locations. ‘De Chi’, an acupuncture sensation reported by acupuncturists and patients, is not required. A retrospective cohort study on postherpetic neuralgia based on a small sample size found pain reduction effects and non-significant differences between the analgesia provided by press tack needles and electro acupuncture [25]. In an animal study, press tack needle was proven to display an anti-stress effect in stress-conditioned rats [26].

### Limitations

The study had serval limitations. The study implemented a telephone call follow-up that caused a significant loss to the 2-weeks and 3-months follow-ups, although missing data were imputed, this might limit the study conclusion on the mid-long-term effect of acupuncture on BCT recovery. When patients reject a measurement due to too much pain, the data was not measured and handled as missing data instead of ranking the pain as maximum. The study only included east Asian patients and only represents this specific population. The Tri-flow measurements only represent 1 aspect of lung function and the study does not present a full investigation on effect of acupuncture on lung function.

## CONCLUSION

This study resulted that press tack needle acupuncture effect on pain reduction in acute BCT patients was inconclusive, as depicted by the FSR score significant analgesic effect, nevertheless, there were no significant differences between the groups over NRS measurements. Subgroup analysis revealed that patients without lung contusion and pleural drainage had a higher likelihood of analgesic effect following press tack needle acupuncture. The application of press tack acupuncture was not associated with changes in sleep quality and lung function. The use of press tacks as a blinding device indicated sufficient blinding levels of patients, and reasonable blinding levels of the acupuncturist. This study verifies that a double-blind design in acupuncture is possible with the use of the press tack needles and placebos. However, future studies on the analgesic effect of acupuncture on BCT patients are warranted to clarify our findings.

## SUPPLEMENTARY MATERIAL

Supplementary material is available at *ICVTS* online.

## Supplementary Material

ivac158_Supplementary_Data

## ABBREVIATIONS

BCT: Blunt chest trauma
FRS: Face rating scale
ISS: Injury severity score
NRS: Numerical rating scale
Tri-flow: Portable incentive spirometer
VAS: Visual analogue scale
