# Supplementary material for: Press tack needle stimulation for blunt chest trauma: a randomized double-blind control trial
Source: Interact Cardiovasc Thorac Surg. 2022 Jun 7;35(1):ivac158. doi: 10.1093/icvts/ivac158 (PMC9204160; doi:10.1093/icvts/ivac158)
Supplement: ivac158_Supplementary_Data [file ivac158_supplementary_data.docx]

**Supplementary materials**

**Supplementary Table 1 – Original data (without missing data imputation)**

|  | **Treatment group** | **N** | **Control group** | **N** | **P-value** |
| --- | --- | --- | --- | --- | --- |
| **Face Rating Scale (FRS)** |  |  |  |  |  |
| Baseline | 2.00±0.89 | 34 | 2.26±0.82 | 35 | 0.279 |
| Immediately after | 1.79±0.81 | 34 | 2.13±0.83 | 32 | 0.049* |
| Day 4 | 1.31±0.81 | 31 | 1.85±0.96 | 34 | 0.020* |
| **NRS score** |  |  |  |  |  |
| ***Pain Rest:*** |  |  |  |  |  |
| Baseline | 3.13±2.10 | 36 | 3.47±2.63 | 36 | 0.538 |
| Immediately after | 2.78±2.25 | 36 | 3.18±2.46 | 36 | 0.471 |
| Day 4 | 1.94±1.73 | 35 | 2.42±1.98 | 36 | 0.307 |
| 2 weeks | 1.96±2.22 | 24 | 2.45±2.23 | 29 | 0.309 |
| 3 months | 0.52±1.31 | 22 | 0.52±1.04 | 28 | 0.432 |
| ***Pain Deep Breath:*** |  |  |  |  |  |
| Baseline | 4.37±2.26 | 35 | 4.57±2.30 | 35 | 0.715 |
| Immediately after | 3.87±2.11 | 35 | 4.17±2.58 | 36 | 0.660 |
| Day 4 | 2.87±1.80 | 34 | 3.38±2.29 | 36 | 0.459 |
| 2 weeks | 1.98±2.28 | 24 | 2.60±2.68 | 29 | 0.420 |
| 3 months | 0.64±1.30 | 21 | 0.36±1.14 | 29 | 0.255 |
| ***Pain Cough:*** |  |  |  |  |  |
| Baseline | 6.32±2.51 | 34 | 6.71±2.77 | 34 | 0.553 |
| Immediately after | 5.92±2.47 | 32 | 6.32±2.83 | 32 | 0.493 |
| Day 4 | 4.58±2.33 | 32 | 5.26±2.91 | 35 | 0.423 |
| 2 weeks | 2.94±2.27 | 24 | 4.17±2.76 | 29 | 0.100 |
| 3 months | 0.84±1.48 | 22 | 0.63±1.64 | 28 | 0.365 |
| ***Pain Body Turn:*** |  |  |  |  |  |
| Baseline | 6.786±2.51 | 35 | 7.278±2.27 | 36 | 0.376 |
| Immediately after | 6.543±2.72 | 35 | 6.125±2.93 | 36 | 0.583 |
| Day 4 | 4.57±2.48 | 34 | 5.57±2.32 | 36 | 0.066 |
| 2 weeks | 3.31±2.79 | 24 | 4.33±2.83 | 29 | 0.184 |
| 3 months | 0.82±1.41 | 22 | 0.59±1.02 | 28 | 0.776 |

**Supplementary Table 2: Number of Patients Refuse to Measure Due to “too much pain”**

| **NRS score** | **Treatment group** | **Control group** |
| --- | --- | --- |
| **Pain Deep Breath:** |  |  |
| Baseline | 1 | 1 |
| Immediately after | 0 | 0 |
| Day 4 | 1 | 0 |
| **Pain Cough:** |  |  |
| Baseline | 2 | 2 |
| Immediately after | 4 | 4 |
| Day 4 | 3 | 1 |
| **Pain Body Turn:** |  |  |
| Baseline | 1 | 0 |
| Immediately after | 0 | 0 |
| Day 4 | 1 | 0 |

Table 3: The table describes the number of patients that rejected the doctor’s request to perform the deep breathing test, to cough or to turn over and lie down on one side as a result of hyperalgesic sensations experienced. NRS - Numeric Rating Scale.

**Supplementary Table 3: Verran Snyder-Halpern Sleep Scale**

|  | Treatment Group | N | Control Group | n | *P-*value |
| --- | --- | --- | --- | --- | --- |
| Sleep Disturbance  Baseline  Day 4 | 300.00±132.53  231.41±123.86 | 35  34 | 287.80±149.59  226.03±132.19 | 35  36 | 0.703  0.685 |
| Sleep Effectiveness  Baseline  Day 4 | 203.64±65.66  232.13±63.05 | 36  35 | 210.17±59.07  241.17±54.36 | 35  36 | 0.800  0.868 |
| Sleep Supplementation  Baseline  Day 4 | 117.11±83.62  87.51±68.27 | 36  35 | 87.74±77.74  90.50±71.80 | 35  36 | 0.062  0.840 |

Table 6: The table describes the findings of the Verran Snyder-Halpern sleep scale. The results were combined into 3 main sections: sleep disturbance, sleep effectiveness and sleep supplementation.

**Supplementary Table 4: Blindness Evaluation**

|  | Correct | Wrong |
| --- | --- | --- |
| Acupuncturist group guess | 34 (58.6%) | 24 (41.4%) |
| Patient’s group guess | 29 (43.9%) | 37 (56.1%) |

Table 7: The table describes the speculations of the patients and acupuncturists if the treatment was using the press tack needles or press tack placebos. There were 14 missing cases in the Acupuncturist group predictions and 6 missing cases in the Patient’s group predictions.

**Supplementary Table 5: CONSORT 2010 checklist of information to include when reporting a randomised trial***

| **Section/Topic** | **Item No** | **Checklist item** | **Reported on page No** |
| --- | --- | --- | --- |
| **Title and abstract** | | | |
|  | 1a | Identification as a randomised trial in the title | 1 |
|  | 1b | Structured summary of trial design, methods, results, and conclusions (for specific guidance see CONSORT for abstracts) | 3 |
| **Introduction** | | | |
| Background and objectives | 2a | Scientific background and explanation of rationale | 4 |
|  | 2b | Specific objectives or hypotheses | 5 |
| **Methods** | | | |
| Trial design | 3a | Description of trial design (such as parallel, factorial) including allocation ratio | 5 |
|  | 3b | Important changes to methods after trial commencement (such as eligibility criteria), with reasons | NA |
| Participants | 4a | Eligibility criteria for participants | 6 |
|  | 4b | Settings and locations where the data were collected | 5 |
| Interventions | 5 | The interventions for each group with sufficient details to allow replication, including how and when they were actually administered | 7 |
| Outcomes | 6a | Completely defined pre-specified primary and secondary outcome measures, including how and when they were assessed | 8 |
|  | 6b | Any changes to trial outcomes after the trial commenced, with reasons | NA |
| Sample size | 7a | How sample size was determined | Published study protocol |
|  | 7b | When applicable, explanation of any interim analyses and stopping guidelines | Published study protocol |
| Randomisation: |  |  |  |
| Sequence generation | 8a | Method used to generate the random allocation sequence | 6 |
|  | 8b | Type of randomisation; details of any restriction (such as blocking and block size) | 7 |
| Allocation concealment mechanism | 9 | Mechanism used to implement the random allocation sequence (such as sequentially numbered containers), describing any steps taken to conceal the sequence until interventions were assigned | 7 |
| Implementation | 10 | Who generated the random allocation sequence, who enrolled participants, and who assigned participants to interventions | 7 |
| Blinding | 11a | If done, who was blinded after assignment to interventions (for example, participants, care providers, those assessing outcomes) and how | 7 |
|  | 11b | If relevant, description of the similarity of interventions | 7 |
| Statistical methods | 12a | Statistical methods used to compare groups for primary and secondary outcomes | 8-9 |
|  | 12b | Methods for additional analyses, such as subgroup analyses and adjusted analyses | Published study protocol |
| **Results** | | | |
| Participant flow (a diagram is strongly recommended) | 13a | For each group, the numbers of participants who were randomly assigned, received intended treatment, and were analysed for the primary outcome | 9/ figure 1 |
|  | 13b | For each group, losses and exclusions after randomisation, together with reasons | 9/ figure 1 |
| Recruitment | 14a | Dates defining the periods of recruitment and follow-up | 9 |
|  | 14b | Why the trial ended or was stopped | N/A |
| Baseline data | 15 | A table showing baseline demographic and clinical characteristics for each group | Table 1 |
| Numbers analysed | 16 | For each group, number of participants (denominator) included in each analysis and whether the analysis was by original assigned groups | 9-11 |
| Outcomes and estimation | 17a | For each primary and secondary outcome, results for each group, and the estimated effect size and its precision (such as 95% confidence interval) | 9-11 |
|  | 17b | For binary outcomes, presentation of both absolute and relative effect sizes is recommended | N/A |
| Ancillary analyses | 18 | Results of any other analyses performed, including subgroup analyses and adjusted analyses, distinguishing pre-specified from exploratory | 9-11 |
| Harms | 19 | All important harms or unintended effects in each group (for specific guidance see CONSORT for harms) | 11 |
| **Discussion** | | | |
| Limitations | 20 | Trial limitations, addressing sources of potential bias, imprecision, and, if relevant, multiplicity of analyses | 12 |
| Generalisability | 21 | Generalisability (external validity, applicability) of the trial findings | 11-14 |
| Interpretation | 22 | Interpretation consistent with results, balancing benefits and harms, and considering other relevant evidence | 11-14 |
| **Other information** | | |  |
| Registration | 23 | Registration number and name of trial registry | 5 |
| Protocol | 24 | Where the full trial protocol can be accessed, if available | 5 |
| Funding | 25 | Sources of funding and other support (such as supply of drugs), role of funders | 15 |

*We strongly recommend reading this statement in conjunction with the CONSORT 2010 Explanation and Elaboration for important clarifications on all the items. If relevant, we also recommend reading CONSORT extensions for cluster randomised trials, non-inferiority and equivalence trials, non-pharmacological treatments, herbal interventions, and pragmatic trials. Additional extensions are forthcoming: for those and for up to date references relevant to this checklist, see [www.consort-statement.org](http://www.consort-statement.org).
